# IGF2BP3 enhances ferroptosis resistance in colon cancer by stabilizing SLC7A11 and is regulated by miR-98-5p

**DOI:** 10.3389/fonc.2025.1576895

**Published:** 2025-06-03

**Authors:** Yaya Sun, Qingwei Liu, Junjie Wu, Guangqing Jiang, Hongzhou Shi, Yihong Zhang, Yanxuan Ling, Weimin Sun, Xin Shi, Congxing Liu

**Affiliations:** ^1^ School of Medicine, Southeast University, Nanjing, China; ^2^ Department of General Surgery, Affiliated Zhongda Hospital of Southeast University, Nanjing, China; ^3^ Department of General Surgery, Xuyi County People’s Hospital, Huai’an, China

**Keywords:** IGF2BP3, colon cancer, ferroptosis, miR-98-5p, SLC7A11

## Abstract

**Purpose:**

Colon cancer remains a global health challenge with rising mortality, necessitating novel therapeutic strategies. IGF2BP3, an oncogenic RNA-binding protein, is implicated in colon cancer progression, while its role in ferroptosis regulation remains unclear. This study investigates IGF2BP3’s role in ferroptosis regulation and its interplay with miR-98-5p in colon cancer, aiming to identify therapeutic targets for enhancing ferroptosis sensitivity.

**Methods:**

Bioinformatics analyses (TCGA, UALCAN, GEPIA) assessed IGF2BP3 expression and prognostic relevance in colon cancer. The expression of IGF2BP3 in clinical samples were analyzed via immunohistochemistry (IHC) and Western blotting. IGF2BP3-knockdown (KD) cell lines (HCT116/DLD-1) were generated using lentiviral shRNA. Ferroptosis sensitivity was evaluated via CCK-8 assays, MDA/ROS quantification, and rescue experiments with ferrostatin-1. Transcriptomic sequencing, RNA immunoprecipitation (RIP), and RNA stability assays identified IGF2BP3-regulated targets. Dual-luciferase reporter assays validated miR-98-5p-IGF2BP3 interactions. Xenograft models assessed in vivo tumor growth.

**Results:**

IGF2BP3 was significantly upregulated in colon cancer tissues and correlated with advanced T-stage, higher clinical stage, and poor survival (p<0.05). Knockdown of IGF2BP3 enhanced erastin-induced ferroptosis sensitivity, marked by elevated MDA and ROS levels, reversible by ferrostatin-1. Mechanistically, IGF2BP3 stabilized SLC7A11 mRNA via direct binding and SLC7A11 overexpression rescued ferroptosis resistance in IGF2BP3-KD cells. miR-98-5p directly targeted IGF2BP3’s 3′-UTR, suppressing its expression and enhancing ferroptosis sensitivity. In vivo, IGF2BP3-KD suppressed tumor growth and reduced Ki67/SLC7A11 expression.

**Conclusion:**

IGF2BP3 drives ferroptosis resistance in colon cancer by stabilizing SLC7A11 mRNA, while miR-98-5p antagonizes this pathway via IGF2BP3 downregulation. Targeting the miR-98-5p/IGF2BP3/SLC7A11 axis offers a promising therapeutic strategy to enhance ferroptosis sensitivity and improve colon cancer outcomes.

## Introduction

Colorectal cancer (CRC) ranks as a leading type of malignant neoplasm affecting the digestive tract. According to GLOBOCAN 2020 statistics, CRC ranks as the third most common cancer globally and is the second leading cause of cancer-related deaths (1). Alarmingly, it is projected that by 2030, the number of new CRC cases will exceed 2.2 million, with approximately 1.1 million deaths (2). The hallmarks of cancer are a set of capabilities acquired by human cells as they transition from a normal state to a tumor growth state, with one of the key characteristics being resistance to cell death (3). Historically, apoptosis was deemed the principal mode of regulated cell death (RCD). However, the discovery of ferroptosis, a non-apoptotic form of cell death identified by Scott J. Dixon in 2012, has provided novel insights into tumor development and therapeutic strategies (4, 5). Accumulating evidence suggests that ferroptosis plays a crucial role in tumor suppression and immune surveillance (5, 6). Multiple tumor suppressors have been identified as key regulators that sensitize cells to ferroptosis, thereby contributing to their antitumor activity. Notably, p53 promotes ferroptosis by inhibiting SLC7A11 transcription, a mechanism contributing to its tumor-suppressive role both *in vitro* and *in vivo* (7, 8).

IGF2BP3, a member of the insulin-like growth factor 2 mRNA-binding protein family, has recently been identified as an m6A reader (9). Structurally, IGF2BP3 contains two RNA recognition motifs (RRMs) and four K homology (KH) domains (10). Through its KH domains, IGF2BP3 recognizes m6A-modified RNA sites, enhancing RNA stability and promoting translational efficiency in an m6A-dependent manner (9). Previous studies have demonstrated that IGF2BP3 regulates multiple oncogenic processes in CRC, including cell proliferation, apoptosis, metastasis, angiogenesis, and resistance to cetuximab (11–14). Moreover, IGF2BP3 has been shown to modulate ferroptosis in lung cancer (15) and liver cancer (16). However, its specific role in regulating ferroptosis in colon cancer cells remains largely unexplored.

MicroRNAs (miRNAs), characterized by their brief, single-stranded RNA structure, typically consist of 19 to 25 nucleotide residues. It is estimated that nearly half of all protein-coding genes are subject to miRNA-mediated regulation (17). Importantly, dysregulation of miRNAs is a critical feature of human cancers, influencing key oncogenic processes such as proliferation, angiogenesis, apoptosis, invasion and metastasis (18–20), as well as drug resistance (21). Notably, miR-98-5p has been reported to be significantly downregulated in CRC cell lines (LOVO, HT-29, HCT15, HCT116, SW116) and hepatocellular carcinoma cell lines (HepG2, Hep3B, LM3, and SMCC7721) compared to normal cells (22, 23). Functionally, miR-98-5p is implicated in the regulation of cell proliferation, metastasis, and drug resistance in tumor cells (24, 25). A recent bioinformatics analysis indicated that the ceRNA axis (LINC02432/hsa-miR-98-5p) is significantly positively correlated with the ferroptosis inhibition gene set score (R = 0.66, p < 0.001) (26). The study further demonstrated that the expression of SLC7A11, a critical regulator of ferroptosis, is negatively correlated with hsa-miR-98-5p expression (26). Despite these findings, its role of miR-98-5p in ferroptosis regulation in colon cancer remains unclear.

The current investigation seeks to delineate the functions of IGF2BP3 and miR-98-5p in the ferroptotic pathway of colon cancer cells. Insights into the mechanisms governing their contribution to ferroptosis could potentially lead to the conception of novel treatment modalities for colon cancer.

## Materials and methods

### Bioinformatics

The study utilized publicly available data from The Cancer Genome Atlas - Colon Adenocarcinoma (TCGA-COAD) dataset (https://portal.gdc.cancer.gov/), comprising 449 colon cancer cases and 41 normal cases, for analyzing the expression of IGF2BP3 in colon cancer. Protein expression levels of IGF2BP3 were analyzed and visualized using the UALCAN database (http://ualcan.path.uab.edu/). Survival outcomes were assessed through overall survival analysis conducted using the GEPIA platform (http://gepia.cancer-pku.cn/). Following IGF2BP3 knockdown (KD), transcriptome sequencing was performed on sh-NC and sh-IGF2BP3 cells. Differentially expressed genes were identified using |FoldChange| > 1.5 and padj < 0.05 as filtering criteria. Gene Ontology (GO) and KEGG pathway enrichment analyses were conducted and visualized using R software. To identify IGF2BP3 targets, transcriptome sequencing data were integrated with crosslinking immunoprecipitation and high-throughput sequencing (CLIP-seq) data from the GEO database (https://www.ncbi.nlm.nih.gov/gds/) and ferroptosis-related genes retrieved from the FerrDb database (http://www.zhounan.org/ferrdb/current/). Transcriptomic co-expression patterns between IGF2BP3 and SLC7A11 were systematically analyzed using GEPIA and TIMER 2.0 (http://timer.comp-genomics.org) platforms (27). To identify miRNAs potentially regulating IGF2BP3, predictions were performed using the TargetScan 8.0 (https://www.targetscan.org/vert_80/), miRDB database (https://mirdb.org/) and miRTarBase database (https://mirtarbase.cuhk.edu.cn/). TargetScan 8.0 was further employed to predict potential binding sites within the IGF2BP3 sequence (28).

### Patients sample

The study analyzed 26 paired colon cancer and adjacent non-tumor tissues, along with 153 additional cancer specimens collected from January 2016 to December 2019. IGF2BP3 expression was evaluated via immunohistochemistry, with patients subsequently divided into high and low expression cohorts for the purpose of correlating these levels with their clinicopathological characteristics and prognostic outcomes. The study excluded individuals who had undergone neoadjuvant chemotherapy or radiotherapy prior to surgery, those with carcinoma *in situ*, non-adenocarcinomatous tumors, appendiceal cancers, as well as cases of metastasis or relapse. Postoperative adjuvant therapies followed standard protocols and dosages. Informed consent was obtained from all patients prior to their enrollment in the study, and ethical authorization was granted by the Independent Ethics Committee for Clinical Research at affiliated Zhongda Hospital of Southeast University.

### Immunohistochemical staining

Paraffin-embedded tissue sections underwent immunohistochemical processing, involving deparaffinization, sequential rehydration through a descending series of ethanol concentrations (100%, 95%, 75%), and antigen retrieval via 0.01 M sodium citrate buffer (pH 6.0). Endogenous peroxidase inhibition was achieved with 3% H_2_O_2_ for 10–20 minutes at 37°C, followed by overnight incubation at 4°C with primary antibodies targeting IGF2BP3(A23295; 1:500; Abclonal, Wuhan, China) or SLC7A11(HA721868; 1:100; HUABIO, Hangzhou, China). Subsequently, sections were treated with HRP-labeled secondary antibodies (ZSGB-BIO, China) at 37°C for 20 minutes, followed by 3,3’-diaminobenzidine (DAB, ZSGB-BIO, China) chromogenic reaction and hematoxylin counterstaining. The Pannoramic SCAN system(3DHISTECH, Hungary) was employed for high-resolution imaging of the stained sections. Semi-quantitative analysis of staining intensity(0: no signal; 1: weak; 2: moderate; 3: strong) and distribution(0: 0–5%; 1: 5–25%; 2: 25–50%; 3: 50–75%; 4: 75–100%) was performed, with the median score serving as the threshold for categorization.

### Cell culture

Human colon cancer cell lines HCT116, DLD-1, RKO, SW480 and SW620 were purchased from Zhong Qiao Xin Zhou Biotechnology (Shanghai, China). The culture conditions for these cells were as follows: HCT116, SW620, and SW480 were maintained in DMEM (meilunbio, China) with supplementation of 10% FBS (Homeland Bio, China) and 1% penicillin/streptomycin (Beyotime, China), within an incubator at 37°C and a 5% CO2 environment. DLD-1 cells were grown in RPMI-1640 medium (meilunbio, China), while RKO cells were cultured in MEM (meilunbio, China); both were supplemented with 10% FBS (Homeland Bio, China) and 1% penicillin/streptomycin (Beyotime, China), and incubated at the same temperature and CO2 concentration as the other cell lines.

### Western blotting analysis

Cellular proteins were isolated from each experimental group using a lysis buffer (P0013, Beyotime, China) enhanced with a protease inhibitor mixture (P1005, Beyotime, China). Protein quantification was achieved with a BCA assay kit (MA0082, Meilunbio, China). A uniform quantity of 20 μg of protein was electrophoretically separated on 4-20% SDS-PAGE gels (ACE Biotechnology, China) and subsequently transferred to 0.45 μm PVDF membranes (Millipore, USA). Membranes were blocked with 5% nonfat milk for an hour at room temperature before being exposed to primary antibodies overnight at 4°C. The primary antibodies employed included IGF2BP3 (A23295; 1:500; Abclonal, Wuhan, China), SLC7A11 (CY7046; 1:1000; Abways, China), and GAPDH (A19056; 1:5000; Abclonal, Wuhan, China). Post incubation, the membranes were washed with TBST and then treated with HRP-linked secondary antibodies (AS014; 1:5000; Abclonal, Wuhan, China) for an hour at room temperature. Protein bands were detected using an ECL kit (Life-iLab, China) and captured on the Tanon luminescence imaging system. The raw western blot images are included in the **Supplementary Materials** section.

### knockdown of IGF2BP3

shRNA targeting human IGF2BP3 (sh-IGF2BP3: 5′-GGCTCAGGGAAGAATTTAT-3’) and a scrambled control sequence were packaged into lentiviruses by GenePharma (Shanghai, China). Lentiviruses (multiplicity of infection, MOI = 10) were used to infect HCT116 and DLD-1 cells in the presence of 8μg/mL polybrene (GenePharma, Shanghai, China). Stable IGF2BP3 KD cell lines (HCT116-sh/DLD-1-sh) and control cell lines (HCT116-scr/DLD-1-scr) were selected using 2μg/mL puromycin (Beyotime, China) for 72 hours. The efficiency of the knockdown was validated through qPCR and Western blot analysis.

### Plasmids and drugs

IGF2BP3-overexpressing plasmids were generated by GenePharma (Shanghai, China). Erastin (SparkJade, China) and ferrostatin-1 (SparkJade, China) were used as treatments in cellular experiments.

### CCK8 assay

The viability of cells was evaluated with the Cell Counting Kit-8 (CCK-8) (MedChemExpress, USA). Cells were plated at a density of 8000 cells per well in 96-well plates and allowed to adhere for 24 hours. Thereafter, the cells were exposed to erastin (SparkJade, China) and Ferrostatin-1 (SparkJade, China) at specified concentrations, followed by a 24-hour incubation period. Then, 100 μL of medium containing 10 μL CCK-8 was added, and the plates were incubated for 2 hours at 37°C in the dark before measuring absorbance at 450 nm with a BioTek microplate reader(BioTek, USA).

### MDA assay

Intracellular MDA concentrations were determined in cells cultured in 6-well plates through the use of a lipid peroxidation MDA assay kit (Beyotime, China), with procedures conducted in accordance with the manufacturer’s guidelines. Protein concentrations were determined using a BCA protein assay kit (Meilunbio, China). The MDA content was normalized to protein concentration to calculate the MDA/protein ratio.

### ROS assay

Following overnight seeding in 6-well plates, cells were subjected to three PBS washes prior to a 30-minute incubation with dihydroethidium (Elabscience, E-BC-F005) at 37°C under 5% CO_2_, in darkness. Subsequent to incubation, the cells were subjected to three rinses with PBS, followed by dissociation and subsequent suspension in 500 μL of PBS. Fluorescence readings were taken at excitation and emission wavelengths of 300 nm and 610 nm, respectively. For cell counting, 20 μL of each cell suspension was loaded into a cell counting plate and analyzed using an automated cell counter(Countstar, China).

### RNA extraction, reverse transcription and quantitative PCR

Total RNA extraction from cells was carried out using Trizol reagent (Vazyme, CAT#R411-01), and cDNA synthesis was achieved with the HiScript III RT SuperMix for qPCR kit (Vazyme, CAT#R323-01). RT-qPCR was conducted in triplicate using the Taq Pro Universal SYBR qPCR Master Mix (Vazyme, Q712-02) on the LineGene 9600 Plus system (BIOER Technologies, Hangzhou, China). GAPDH served as the internal control for gene expression normalization. Relative RNA expression levels were determined by the 2^-ΔΔCt method. The sequences of the primers employed in the RT-qPCR are detailed in **Supplementary Table S2**.

### RNA immunoprecipitation

The RNA immunoprecipitation (RIP) was performed utilizing the BersinBio RNA Immunoprecipitation Kit (Cat#Bes5101), adhering to the manufacturer’s recommended protocol. Cell lysates were allocated into three distinct fractions: 0.8 mL for the IP group, 0.8 mL for the IgG group, and 0.1 mL for the Input group. The IP and IgG groups were incubated overnight at 4°C with 5 μg of anti-IGF2BP3 antibody (ab313556; Abcam, UK) or IgG antibody, respectively. The next day, 20 µL of protein A/G magnetic beads were added to each sample and incubated for 1 hour at 4°C. After collection of the magnetic beads with a Millipore magnetic stand, the RNA-protein complexes were eluted. The RNA was then extracted and purified, followed by the quantification of the target RNA levels through RT-qPCR.

### RNA stability assay

The stability of SLC7A11 mRNA was evaluated by treating cells with Actinomycin D (MedChemExpress) at a concentration of 5 μg/mL for 0, 3, and 6 hours. Total RNA was extracted using Trizol reagent (Vazyme, CAT#R411-01) at each time point, and the expression of SLC7A11 mRNA was determined via RT-qPCR. The mRNA degradation rate and half-life were calculated based on established methodologies (9).

### Dual−luciferase reporter assay

To evaluate the interaction between miR-98-5p and the 3′-untranslated region (3′-UTR) of IGF2BP3, wild-type (WT) and mutant (MUT) IGF2BP3 sequences predicted to bind miR-98-5p were amplified and cloned into pmiRGLO vectors (GenePharma). HEK293T cells were planted in 12-well plates and cultured overnight at 37°C. On the subsequent day, they were cotransfected with 50 pmol (100 nM) miR-98-5p mimics and 1 μg of plasmids carrying the WT or MUT IGF2BP3 3’ UTR using GP-transfect-Mate (GenePharma). After 48 hours, the luciferase assay was conducted using the Dual Luciferase Reporter Gene Assay Kit (GenePharma). The efficiency of binding was evaluated by normalizing the firefly luciferase activity against the renilla luciferase activity.

### Subcutaneous xenografts of nude mice

Ten five-week-old BALB/c-nude mice were acquired from Changzhou Cavens Experimental Animal Co. Ltd. and randomly divided into two groups: HCT116-shNC and HCT116-shIGF2BP3. Each mouse received a subcutaneous injection of 3 × 10^6^ HCT116-shNC or HCT116-shIGF2BP3 cells in 200 μL of PBS into the dorsal flanks. Tumor growth was monitored every three days commencing one week following injection by measuring the long (L) and short (S) diameters with a vernier caliper, and tumor volumes were estimated using the formula V= L*S^2^/2. Following three weeks, the mice were humanely euthanized, and the tumors were removed, weighed, and documented photographically. Tumor tissue samples were analyzed by immunohistochemistry (IHC) to assess the expression of IGF2BP3, SLC7A11 and Ki67. The Institutional Ethics Committee of Southeast University granted approval for all animal procedures.

### Statistical analysis

Statistical analyses were conducted by using SPSS 26 (IBM, Armonk, NY, USA) and GraphPad Prism 9 (GraphPad, La Jolla, CA, USA). Data are represented as the mean values ± standard deviation, derived from three repetitions of the experiments. Statistical inference was made using Student’s t-test and one-way ANOVA. A two-tailed p-value less than 0.05 was deemed to indicate statistical significance.

## Results

### IGF2BP3 is upregulated in colon cancer and is a potential biomarker for prognosis

**Figure 1:**
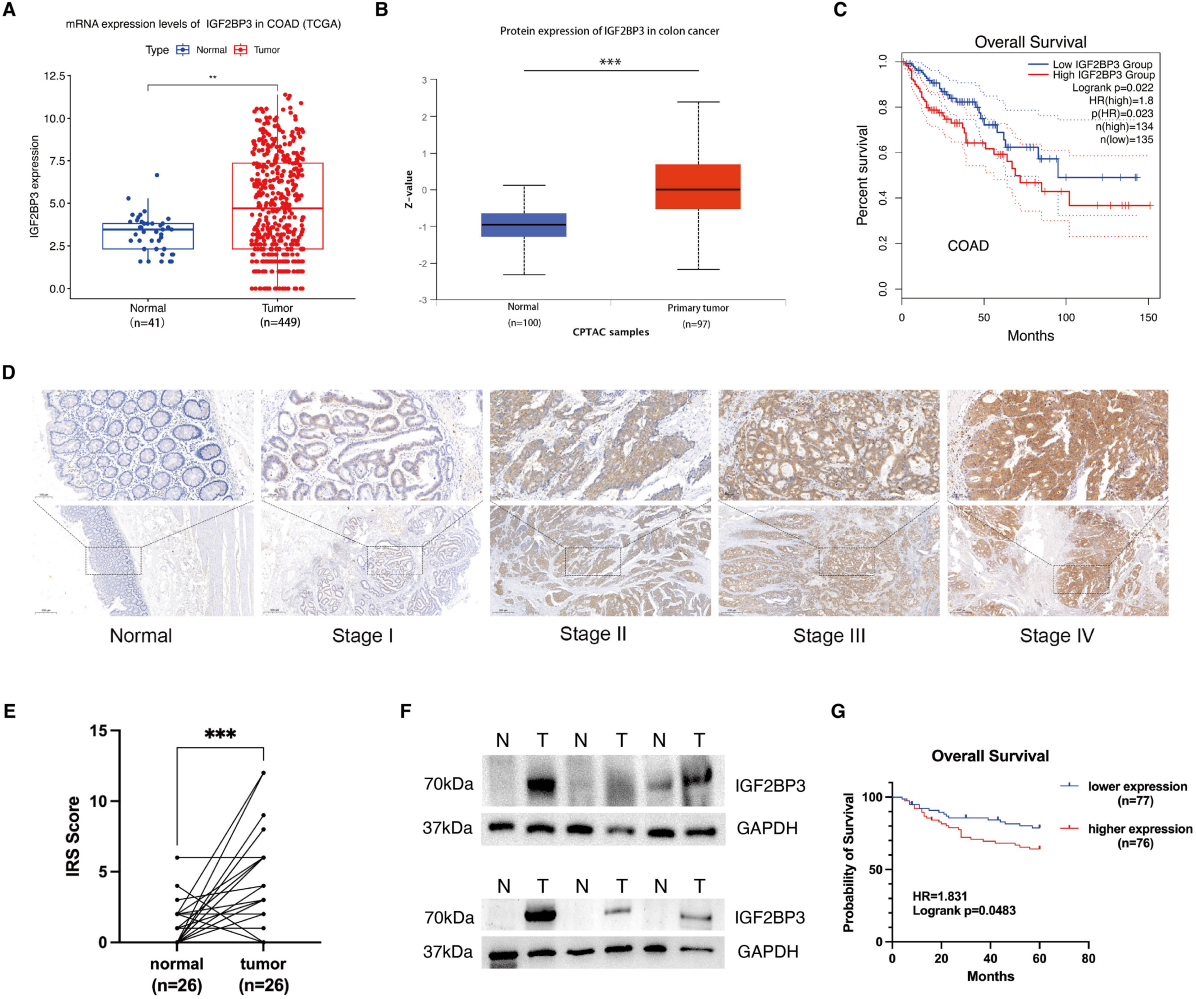
IGF2BP3 is upregulated in colon cancer and high IGF2BP3 expression indicates poor prognosis. **(A)** Expression of IGF2BP3 in paired adjacent–tumorous tissues in COAD patients in TCGA database. **(B)** Expression of IGF2BP3 in normal and COAD tissues as analyzed using the UALCAN database. **(C)** Overall survival curves obtained from GEPIA database based on IGF2BP3 expression in COAD patients. **(D)** Representative IHC images of IGF2BP3 in normal tissues and tumorous tissues from different TNM stages. **(E)** Statistics of the IGF2BP3 IHC scores from 26 pairs of tumor and adjacent normal tissues. **(F)** WB analysis of six pairs of fresh tumor and adjacent normal tissue samples. **(G)** Overall survival curves based on IGF2BP3 expression in 153 colon cancer patients in Zhongda Hospital Affiliated to Southeast University. Statistical analysis was performed using t-test **(A, B)**, or paired t-test ANOVA **(E)**. **P < 0.01, ***P < 0.001.

This study embarked on a thorough investigation of IGF2BP3 expression by examining various datasets and employing a range of experimental strategies. The analysis of the TCGA database revealed a substantial increase in IGF2BP3 mRNA expression in colon cancer samples, when compared with those from normal colonic mucosa, as represented in **Figure 1A**. This finding was corroborated by the UALCAN database, which indicated an increase in IGF2BP3 protein levels within the colon cancer tissues (**Figure 1B**). Moreover, the GEPIA database analysis indicated that patients with colon cancer who exhibited high levels of IGF2BP3 expression were correlated with an unfavorable prognosis (**Figure 1C**). To validate these findings, immunohistochemical (IHC) analysis was performed on 26 sets of tumor tissues and their adjacent normal tissue counterparts, revealing that IGF2BP3 was predominantly overexpressed in tumor tissues (**Figures 1D, E**). To confirm these results further, six matched sets of freshly procured tumor tissues and their adjacent normal tissue samples were gathered, and the expression of IGF2BP3 was evaluated through the implementation of Western blotting. The data indicated that the majority of tumors displayed a marked overexpression of IGF2BP3 relative to adjacent normal tissue (**Figure 1F**). Then, we extended IHC analysis to a cohort of 153 colon cancer cases with detailed clinicopathological and follow-up data. The upregulated expression of IGF2BP3 was found to be intimately linked with a more advanced T classification, a higher clinical stage, and a diminished overall survival rate, which collectively underscores its potential utility as a pivotal prognostic biomarker (**Table 1**; **Figure 1G**). These cumulative results highlight the increased expression of IGF2BP3 in colon cancer, suggesting a potential link to a less favorable prognosis.

**Table 1:** Relationship between IGF2BP3 expression and the clinicopathological features in colon cancer patients.

Pathological characteristics	Low expression (n=77)	High expression (n=76)	X^2^ value	P value
TNM stage Stage I Stage II Stage III Stage IV	12(15.6%) 35(45.5%) 27(35.1%) 3(3.9%)	3(3.9%) 30(39.5%) 35(46.1%) 8(10.5%)	9.083	0.028^*^
T stage T_1_ T_2_ T_3_ T_4_	4(5.2%) 8(10.4%) 57(74.0%) 8(10.4%)	0(0.0%) 4(5.3%) 58(76.3%) 14(18.4%)	8.564	0.036^*^
N stage N**_0_** N**_1-2_**	47(61.0%) 30(39.0%)	32(42.1%) 44(57.9%)	5.490	0.019^*^
M stage M**_0_** M**_1_**	74(96.1%) 3(3.9%)	68(89.5%) 8(10.5%)	2.520	0.112
Differentiation Low grade High grade	67(87.0%) 10(13.0%)	62(81.6%) 14(18.4%)	0.854	0.355
Nerve invasion No Yes	62(80.5%) 15(19.5%)	55(72.4%) 21(27.6%)	1.412	0.235
Vascular invasion No Yes	59(76.6%) 18(23.4%)	50(65.8%) 26(34.2%)	2.191	0.139
MMR state pMMR dMMR	66(85.7%) 11(14.3%)	66(86.8%) 10(13.2%)	0.041	0.839

IGF2BP3, Insulin-like growth factor 2 mRNA-binding protein 3; MMR, Mismatch repair; pMMR, Proficient mismatch repair; dMMR, Deficient mismatch repair. *P < 0.05.

### IGF2BP3 deficiency promotes ferroptosis in colon cancer cells

**Figure 2:**
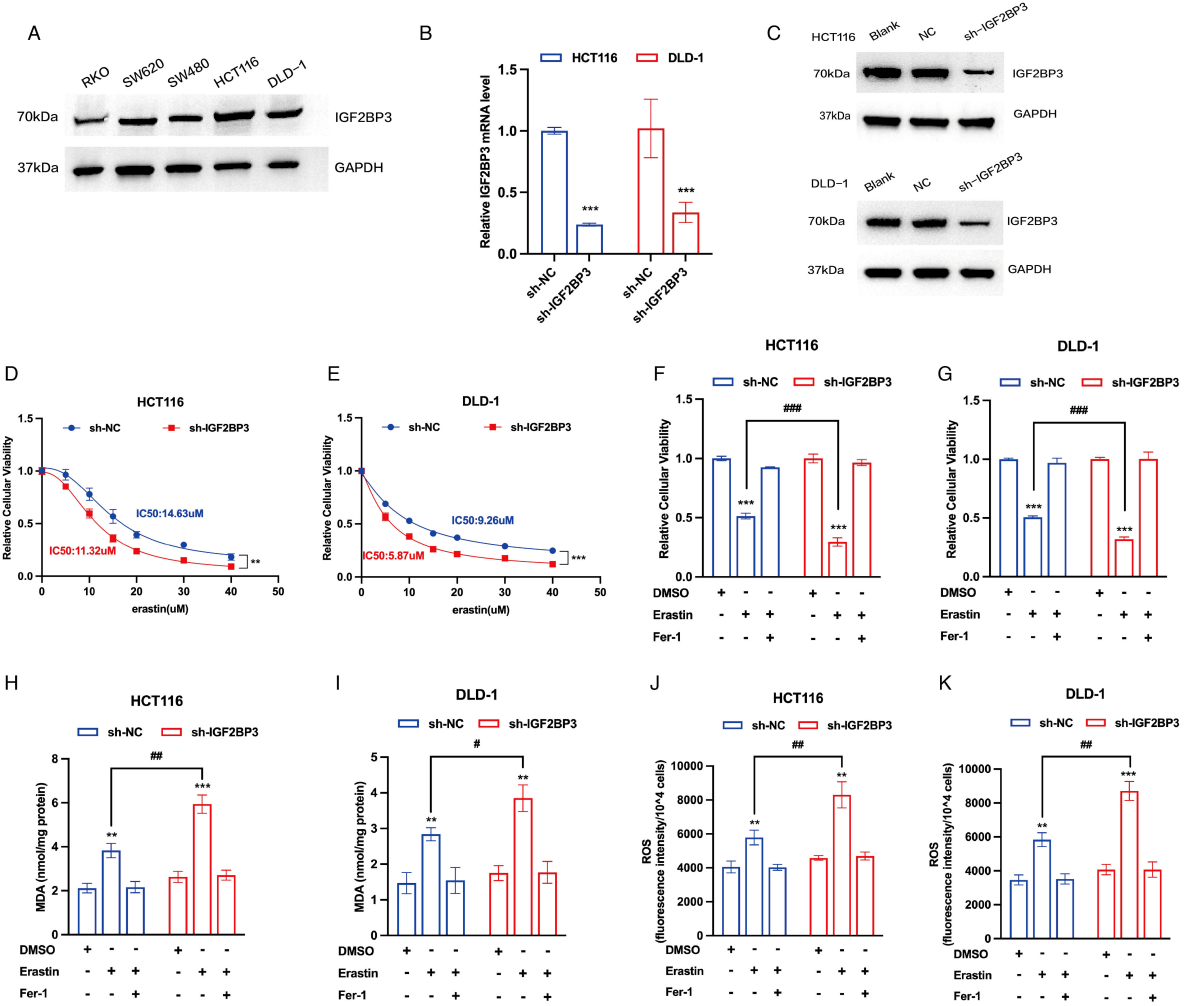
IGF2BP3 deficiency promotes ferroptosis in colon cancer cells. **(A)** The expression of IGF2BP3 in various colon cancer cell lines performed by Western Blotting analysis. **(B,C)** Knockdown of IGF2BP3 in HCT116 and DLD-1 confirmed by qPCR and Western Blotting analysis. **(D,E)** Viability curves of HCT116 and DLD-1 cells with or without IGF2BP3 knockdown treated with erastin for 24h at the indicated concentrations. **(F-K)** Relative cellular viability **(F,G)**, MDA levels **(H,I)** and intracellular ROS levels **(J,K)** of HCT116 and DLD-1 cells with or without IGF2BP3 knockdown under the indicated conditions with treatment of erastin (15 μM, 24 h; F,H,J) or erastin (10 μM, 24 h; G,I,K). HCT116 and DLD-1 cells were also treated with or without ferrostatin-1 (2 μM, 24 h). All experiments were repeated three times, and the data are shown as the mean ± SD. Statistical analysis was performed using t-test. **P < 0.01, ***P < 0.001. #P < 0.05, ##P < 0.01, ###P < 0.001.

To select the appropriate cell line for IGF2BP3 knockdown, our initial investigation involved measuring the IGF2BP3 expression in a panel of five colon cancer cell lines. HCT116 and DLD-1, characterized by their comparatively high levels of IGF2BP3 expression, were chosen for subsequent phenotypic experiments (**Figure 2A**). To elucidate the potential role of IGF2BP3 in the modulation of ferroptosis within colon cancer, we created stable cell lines by employing lentiviral vectors to mediate the knockdown of IGF2BP3 in both HCT116 and DLD-1 cells (**Figures 2B, C**). To delve into the connection between IGF2BP3 and ferroptosis, two IGF2BP3 KD cell lines were treated with varying concentrations of erastin. The results indicated that a deficiency in IGF2BP3 markedly enhanced the susceptibility of colon cells to erastin-triggered cell death (**Figures 2D, E**). Furthermore, a CCK-8 assay indicated that erastin-induced cell death was augmented in IGF2BP3-knockout cells, an effect that was considerably rescued by the ferroptosis inhibitor ferrostatin-1 (**Figures 2F, G**). These findings suggest that IGF2BP3 knockdown sensitized CRC cells to erastin-induced cell death. Additional analyses were performed to examine the accumulation of malondialdehyde (MDA) levels and reactive oxygen species (ROS) levels. The findings revealed that cells with IGF2BP3 deficiency exhibited a marked increase levels in MDA (**Figures 2H, I**) and ROS (**Figures 2J, K**), and these effects were reversed upon treatment with ferrostatin-1. Together, the collected evidence indicates that IGF2BP3 deficiency increases the vulnerability of colon cancer cells to ferroptosis.

### Knockdown of IGF2BP3 repressed tumor growth *in vivo*

**Figure 3:**
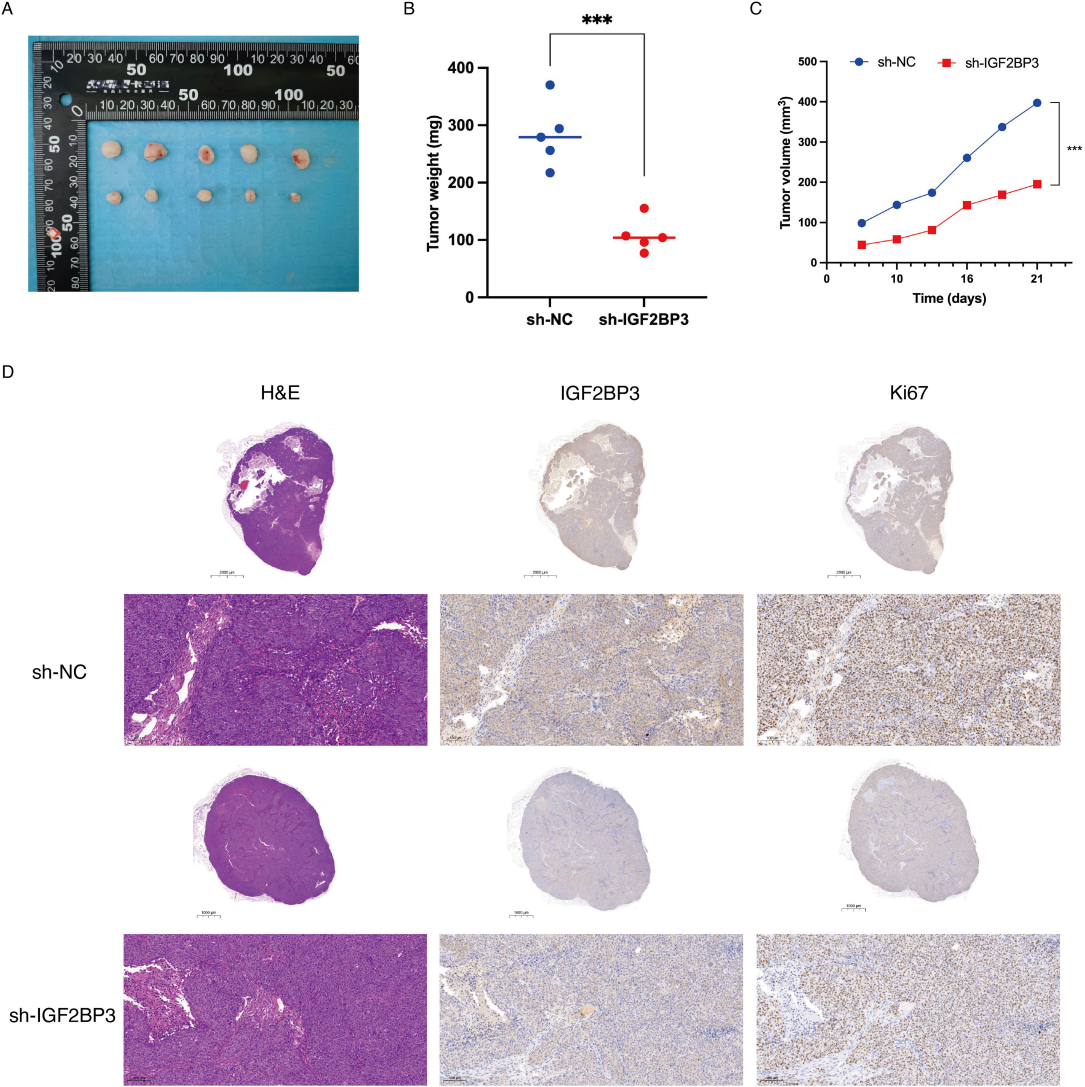
Knockdown of IGF2BP3 repressed tumor growth *in vivo*. **(A)** Subcutaneous tumors were observed at 21 days in two different groups (n=5) and the excised tumors were photographed. **(B)** Nude mice were sacrificed, and xenografts were harvested and weighed. **(C)** Tumor growth curve after the injection of IGF2BP3 knockdown HCT116 cells and control cells into nude mice. **(D)** Representative images of H&E and IHC staining for xenografts derived from HCT116 cells. IHC staining was performed for the detection of IGF2BP3 and Ki67 expression in the xenografts.

Then, we assessed the *in vivo* effects of IGF2BP3 knockdown on tumor progression via the application of a xenograft model. Consistent with our *in vitro* findings, knockdown of IGF2BP3 significantly suppressed xenograft growth in nude mice, which was demonstrated by a decrease in both tumor volume and tumor weight (**Figures 3A-C**). Furthermore, immunohistochemical analysis revealed that the downregulation of IGF2BP3 resulted in a significant decrease in Ki67 expression, a recognized indicator of tumor proliferation (**Figure 3D**). The findings imply that IGF2BP3 is essential for promoting tumor growth *in vitro*.

### IGF2BP3 regulates ferroptosis in colon cancer cells through its interaction with SLC7A11

**Figure 4:**
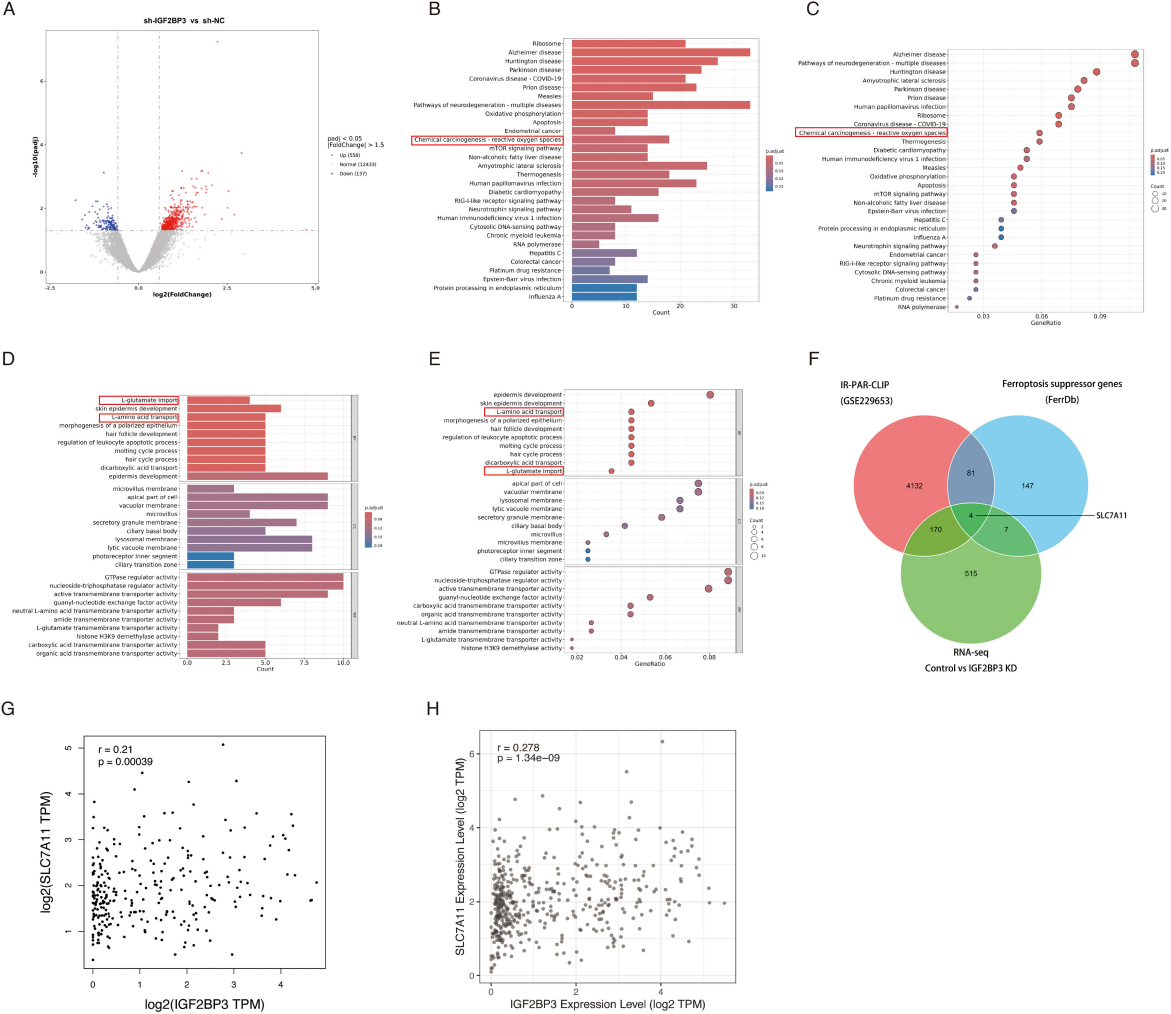
Identification of DEGs and the pathway enrichment analysis between negative control and IGF2BP3 knockdown subgroups. **(A)** Volcano plot of the DEGs expression between RSL1D1 high and low subgroups. The blue and red dots represented the significantly downregulated and upregulated genes, respectively; The black dots represented the genes without differential expression. **(B-E)** Results of GO and KEGG enrichment analysis for DEGs. The X-axis represents count or gene ratio and the Y-axis represents different enriched pathways. **(F)** The 4 IGF2BP3 mRNA targets, as revealed by comparing IR-PAR-CLIP, RNA-seq, and ferroptosis suppressor genes from FerrDb database. **(G,H)** IGF2BP3 and SLC7A11 co-expression patterns in GEPIA **(G)** and TIMER **(H)** databases.

To delve deeper into the underlying mechanisms through which IGF2BP3 governs ferroptosis in colon cancer cell, we performed transcriptomic sequencing on three pairs of negative control and IGF2BP3 KD cells. The volcano plot revealed differentially expressed genes following IGF2BP3 knockdown (**Figure 4A**). Subsequently, we conducted Gene Ontology (GO) and Kyoto Encyclopedia of Genes and Genomes (KEGG) pathway enrichment analyses on these differentially expressed genes (**Figures 4B-E**). KEGG analysis indicated that IGF2BP3 participates in modulating the reactive oxygen species (ROS) signaling pathway in colorectal cancer (**Figures 4B, C**). GO analysis elucidated that IGF2BP3 contributes to the cellular process of amino acid transport, particularly glutamate transport (**Figures 4D, E**). Ferroptosis is a unique form of cell death driven by iron-dependent lipid peroxidation, which involves the reaction of divalent iron with phospholipids containing polyunsaturated fatty acid peroxides (PUFAs-OOH) to generate ROS (6). Amino acid transport, especially the transport of cystine and glutamate, plays a crucial role in regulating the levels of PUFAs-OOH, thereby modulating ferroptosis (29, 30). The presented findings bolster the argument that IGF2BP3 is an active participant in the ferroptotic pathway within colon cancer cells. As IGF2BP3 is known to be an RNA-binding protein, we endeavored to identify the specific mRNAs that it targets and regulates. To investigate this, we undertook a thorough investigation of IR-PAR-CLIP-seq data utilizing anti-IGF2BP3 antibodies (GSE229653), integrated our previously obtained transcriptomic sequencing data, and examined the dataset of ferroptosis suppressor genes from the FerrDb database. Ultimately, we identified that IGF2BP3 may regulate ferroptosis in colon cancer cells through its interaction with SLC7A11 (**Figure 4F**). Subsequently, we investigated the co-expression relationship between IGF2BP3 and SLC7A11 in public databases. Analysis of GEPIA database revealed a statistically significant positive correlation between IGF2BP3 expression and SLC7A11 levels (r = 0.21, p < 0.001) (**Figure 4G**). Similarly, interrogation of the TIMER database confirmed this association, with IGF2BP3 expression demonstrating a significant positive correlation with SLC7A11 expression (r = 0.278, p < 0.001) (**Figure 4H**).

**Figure 5:**
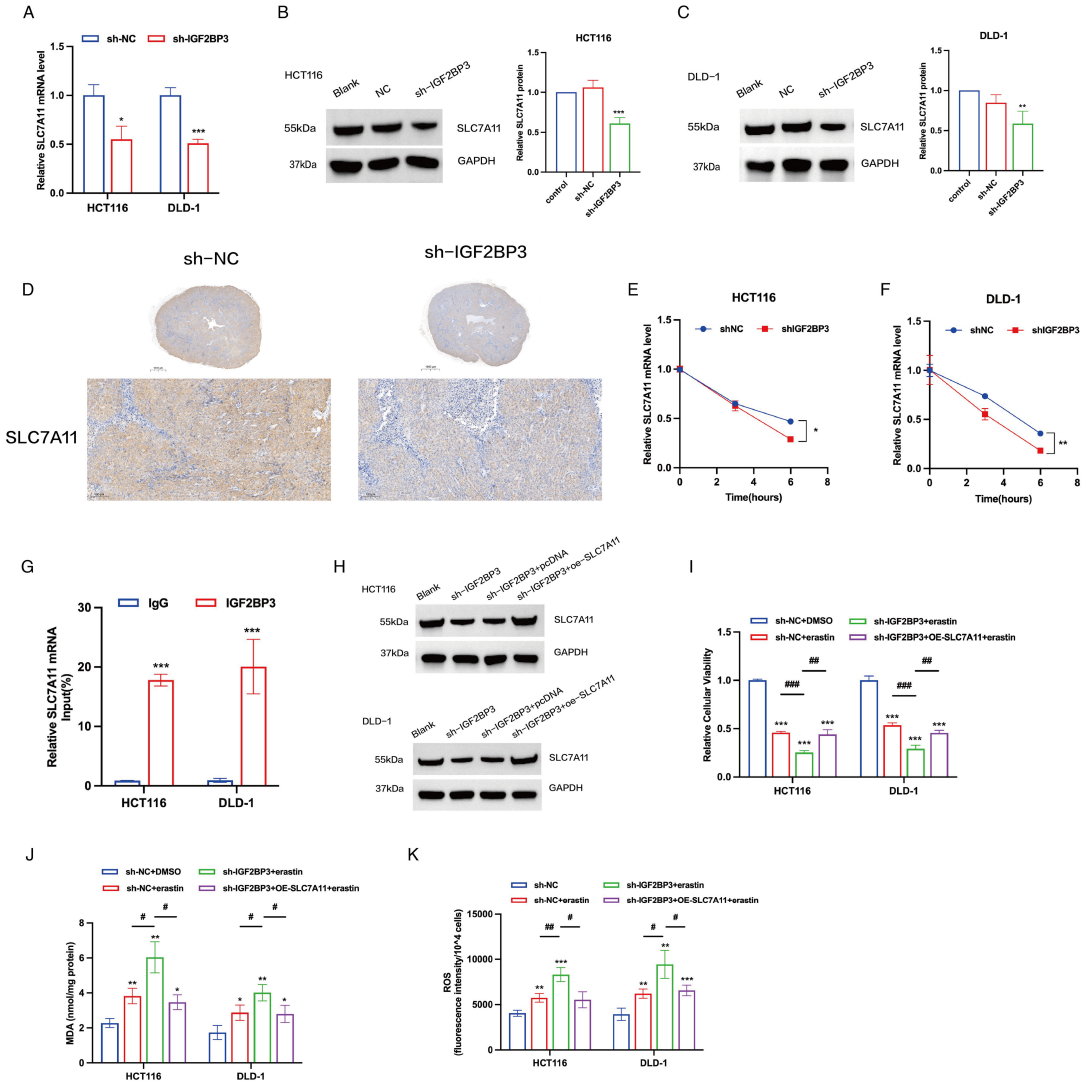
IGF2BP3 regulates ferroptosis in colon cancer cells by regulating the stability of SLC7A11 mRNA. **(A)** mRNA expression levels of SLC7A11 in control cells and cells with IGF2BP3 knockdown. **(B, C)** Representative WB images of SLC7A11 in control cells and cells with IGF2BP3 knockdown. **(D)** IHC staining was performed for the detection of SLC7A11. **(E, F)** The mRNA stability and degradation halftime of SLC7A11 in HCT116 and DLD-1 treated by Actinomycin D. **(G)** RIP experiments using control IgG and anti-IGF2BP3 antibodies to examine IGF2BP3 binding to SLC7A11 mRNA. **(H)** Representative WB images of SLC7A11 in control cells, cells with IGF2BP3 knockdown and cells with SLC7A11 overexpression. **(I-K)** Relative cellular viability **(I)**, MDA levels **(J)** and intracellular ROS levels **(K)** of HCT116 and DLD-1 cells under the indicated conditions with treatment of erastin (15 μM, 24 h; HCT116) or erastin (10 μM, 24 h; DLD-1). All experiments were repeated three times, and the data are shown as the mean ± SD. Statistical analysis was performed using t-test **(A-C, G, I-K)**, or two-way ANOVA **(E, F)**. *P < 0.05, **P < 0.01, ***P < 0.001. #P < 0.05, ##P < 0.01, ###P < 0.001.

To determine whether IGF2BP3 regulates the identified target proteins, we compared the expression level of SLC7A11 before and after IGF2BP3 knockdown in HCT116 and DLD-1 cells. A notable decline in the mRNA expression of SLC7A11 was detected in colon cancer cells following the knockdown of IGF2BP3 (**Figure 5A**). In addition, the protein levels of SLC7A11 mirrored the mRNA expression trends, being positively regulated by IGF2BP3 (**Figures 5B, C**). Consistent with these findings, immunohistochemical analysis of xenograft tumor demonstrated that IGF2BP3 knockdown significantly reduced SLC7A11 expression (**Figure 5D**). Then, we investigated the role of IGF2BP3 in SLC7A11 mRNA stability by treating cells with Actinomycin D (ActD), a transcription inhibitor that prevents the synthesis of new mRNA molecules. Our study revealed that the SLC7A11 mRNA in cells with IGF2BP3 knockdown underwent a quicker degradation rate relative to control cells (**Figures 5E, F**), which points to IGF2BP3’s involvement in the preservation of SLC7A11 mRNA stability. Further supporting this, RNA immunoprecipitation (RIP) analysis demonstrated that SLC7A11 mRNA was significantly enriched in the RIP products pulled down by anti-IGF2BP3 antibodies, compared to the control pulled down by anti-IgG antibodies (**Figure 5G**). This suggests that IGF2BP3 specifically binds to SLC7A11 mRNA. Subsequently, we overexpressed SLC7A11 in IGF2BP3 KD cells by transfecting with a plasmid carrying SLC7A11 (**Figure 5H**). The sensitivity of these cells to erastin was significantly reduced following SLC7A11 overexpression (**Figure 5I**). Additionally, following the overexpression of SLC7A11 in IGF2BP3 knockdown cells that were exposed to erastin, we noted a pronounced attenuation of oxidative stress, which was manifested by a considerable reduction in both MDA and ROS levels (**Figures 5J, K**). In conclusion, these findings suggest that the suppression of IGF2BP3 in colon cancer cells drives ferroptosis through the modulation of SLC7A11 mRNA stability, consequently increasing the susceptibility to ferroptosis.

### miR-98-5p acts as an upstream regulator of IGF2BP3

**Figure 6:**
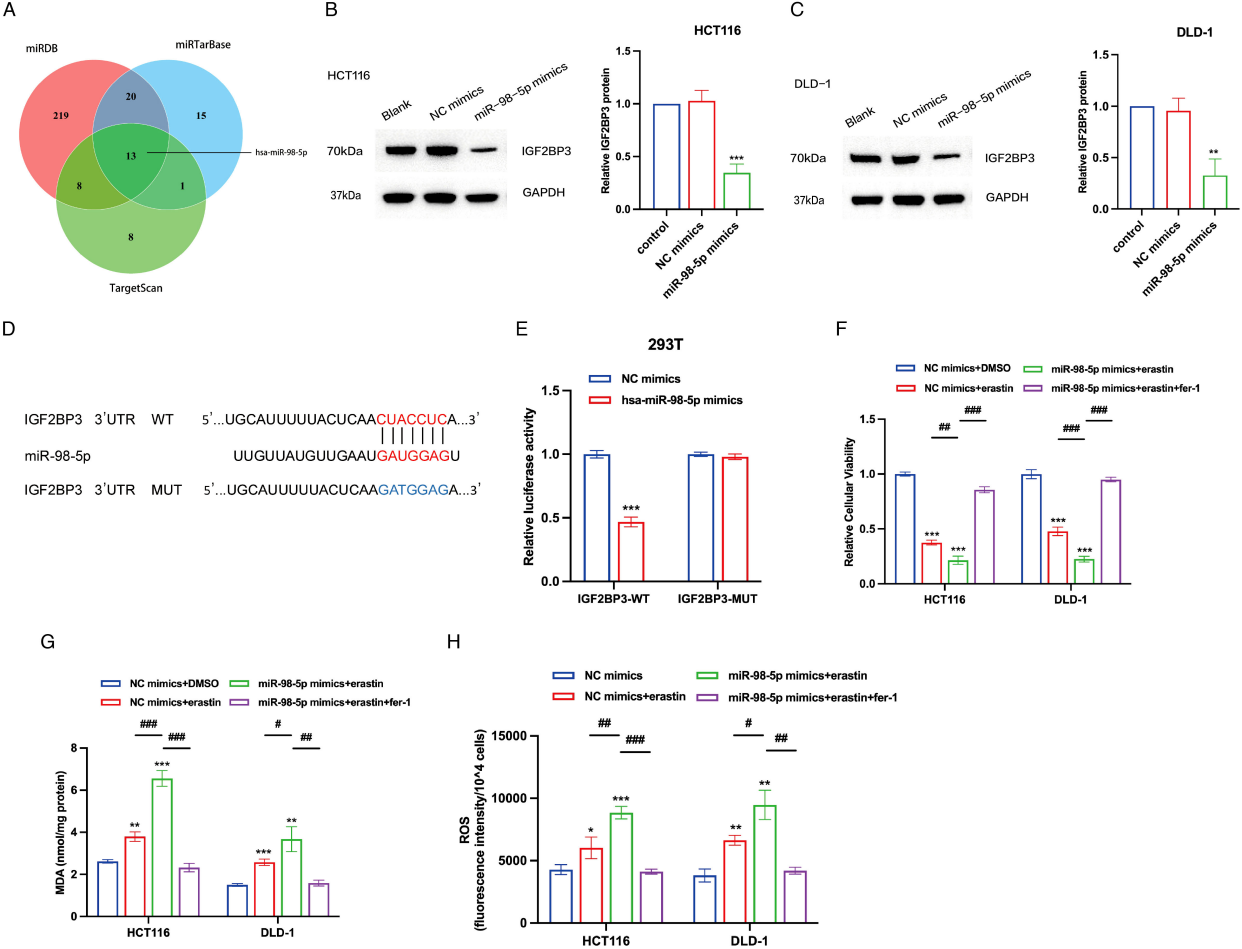
IGF2BP3 is a direct target of miR-98-5p. **(A)** Venn diagram showing the overlap microRNAs from three microRNA prediction algorithms. **(B,C)** Western blotting analysis of IGF2BP3 protein levels in HCT116 and DLD-1 cells treated with miRNA-98-5p mimics or NC mimics. Right panel shows the statistical analysis of the western blot. **(D)** An illustration of the predicted binding site for miR-98-5p in the 3′-UTR of IGF2BP3. **(E)** Luciferase activity assays showing the direct binding efficiency of miR-98-5p and its putative IGF2BP3 3′-UTR target. **(F-H)** Relative cellular viability **(F)**, MDA levels **(G)** and intracellular ROS levels **(H)** of HCT116 and DLD-1 cells under the indicated conditions with treatment of erastin (15 μM, 24 h; HCT116) or erastin (10 μM, 24 h; DLD-1). HCT116 and DLD-1 cells were also treated with or without ferrostatin-1 (2 μM, 24 h). All experiments were repeated three times, and the data are shown as the mean ± SD. Statistical analysis was performed using t-test. *P < 0.05, **P < 0.01, ***P < 0.001. #P < 0.05, ##P < 0.01, ###P < 0.001.

MicroRNAs (miRNAs) serve as essential modulators influencing the processes of cancer initiation and progression (31). To investigate potential miRNAs that regulate IGF2BP3, we utilized the TargetScan, miRTarBase, and miRDB databases to screen for miRNAs that can target IGF2BP3, identifying 13 candidate miRNAs (**Figure 6A**). Literature research indicated that miR-98-5p might act as an upstream regulator, targeting IGF2BP3 and participating in the ferroptosis process in colon cancer cells. miR-98-5p is notably downregulated in colorectal cancer cell lines (LOVO, HT-29, HCT15, HCT116, SW116) compared to normal cells (22). Additionally, bioinformatics analysis suggested that the ceRNA (LINC02432/hsa-miR-98-5p) risk score was significantly positively correlated with the ferroptosis inhibitory gene set score and SLC7A11 expression is negatively correlated with hsa-miR-98-5p expression (26). Furthermore, previous studies had shown that miR-98-5p could target IGF2BP1 and IGF2BP2 to regulate tumor cell growth (23, 32). To ascertain whether miR-98-5p modulates the expression of IGF2BP3, we conducted transfections of miR-98-5p mimics into HCT116 and DLD-1 cells. Notably, miR-98-5p robustly suppressed IGF2BP3 protein levels (**Figures 6B, C**). Utilizing TargetScan, we compared the sequence of miR-98-5p with the 3′-UTR of IGF2BP3 to identify the putative miR-98-5p binding site within the IGF2BP3 3′-UTR (**Figure 6D**). To validate this interaction, we generated a luciferase reporter vector with either the wild-type (WT) or a mutated (MUT) version of the IGF2BP3 3′-UTR. Subsequent cotransfection of 293T cells with the WT IGF2BP3 3′-UTR reporter plasmid and miR-98-5p mimics resulted in a significant reduction in luciferase activity (**Figure 6E**). However, when the MUT IGF2BP3 3′-UTR plasmid was cotransfected with miR-98-5p mimics into 293T cells, there was no significant change in luciferase activity observed (**Figure 6E**), thereby affirming the specific targeting interaction between miR-98-5p and the IGF2BP3 3′-UTR. We next investigated the impact of miR-98-5p on ferroptosis in colon cancer cells. Utilizing the CCK-8 assay, we found that the introduction of miR-98-5p mimics enhanced the susceptibility of colon cancer cells to erastin-induced cytotoxicity, an effect that was reversible with the ferroptosis inhibitor ferrostatin-1 (**Figure 6F**). Additionally, we assessed the levels of MDA and ROS in miR-98-5p-transfected cells. The data revealed that miR-98-5p overexpression led to an elevation in both MDA and ROS levels, and this influence was attenuated following treatment with ferrostatin-1, restoring normal MDA and ROS levels (**Figures 6G, H**). Overall, our results provide strong evidence that miR-98-5p directly binds to the 3′-UTR of IGF2BP3, downregulating its translation, and thereby promoting ferroptosis in colon cancer cells.

## Discussion

Cancer is defined by the acquisition of traits that enable cells to transition from normal to tumorigenic, with resistance to cell death being a key feature (3). Historically, apoptosis was considered the primary form of regulated cell death (RCD). However, with advances in the study of tumor cell biology, several additional subtypes of RCD have been identified, including necrosis, pyroptosis, ferroptosis, and cuproptosis (33–35). Since the discovery of ferroptosis in 2012 by Scott J. Dixon as a novel, non-apoptotic form of cell death, its role in cancer progression and potential therapeutic applications have garnered increasing attention (36, 37).

Ferroptosis is morphologically characterized by a reduction in mitochondrial volume, rupture of the mitochondrial outer membrane, loss or absence of mitochondrial cristae, and unchanged nuclear size with a loss of chromatin staining (38). The heightened focus on ferroptosis in the realm of oncology is attributed to its acknowledged role as an endogenous tumor suppressor process, along with its therapeutic potential for bolstering antitumor immunity. A growing body of evidence strongly supports the concept that ferroptosis functions as a natural antitumor mechanism, exerting tumor-suppressing effects through interactions with various tumor suppressor genes, specific oncogene mutations, and cancer stem cells (CSCs) (36). For example, p53 plays a crucial role in inhibiting tumor growth and promoting ferroptosis, in part by downregulating the expression of SLC7A11, which is achieved either by directly binding to the SLC7A11 promoter or by interacting with ubiquitin-specific peptidase 7 to reduce histone H2B monoubiquitination on the SLC7A11 promoter (39, 40). Moreover, inhibition or genetic knockout of SLC7A11 significantly impedes the growth of KRAS-mutant tumors, highlighting its essential role in resisting ferroptosis to enable the evasion and the subsequent growth of KRAS-driven tumors (41, 42). Additionally, lung CSCs promote SLC7A11 transcription through the upregulation of the SRY-box transcription factor 2, which confers resistance to ferroptosis (43). Apoptosis serves as a defense against cancer, but cancer cells often evade it due to their inherent capabilities and therapy resistance, making it important to explore non-apoptotic forms of regulated cell death (RCD) as potential therapies. Inducing ferroptosis not only inhibits tumor growth but also shows promise for enhancing immunotherapy responses and overcoming resistance to existing cancer treatments (37).

The data obtained from our study suggest that IGF2BP3 is upregulated in colon cancer tissue samples and is associated with unfavorable clinical prognostic indicators. Silencing IGF2BP3 in colon cancer cell lines enhances their sensitivity to ferroptosis, an effect that can be reversed by the ferroptosis inhibitor ferrostatin-1. Mechanistically, IGF2BP3 promotes ferroptosis by stabilizing the mRNA of SLC7A11, a key ferroptosis regulator, through direct interaction with the mRNA. Although IGF2BP3’s involvement in ferroptosis regulation has been documented in both lung and liver cancers, recent studies emphasize its cancer type-specific regulatory mechanisms mediated by divergent downstream targets (15, 16). In hepatocellular carcinoma, IGF2BP3 stabilizes NRF2 mRNA to inhibit ferroptosis; whereas in lung adenocarcinoma, it modulates multiple anti-ferroptotic regulators such as GPX4, SLC3A2, ACSL3, and FTH1 to confer ferroptosis resistance. Despite these findings, our study has several limitations that should be acknowledged. Firstly, although rescue experiments targeting SLC7A11 demonstrated that IGF2BP3 exerts its regulatory role in ferroptosis through this gene, it remains plausible that IGF2BP3 may concurrently or alternatively regulate ferroptosis via other downstream targets. Secondly, while this study provides evidence supporting IGF2BP3’s role in stabilizing SLC7A11 mRNA, the precise molecular mechanisms governing this interaction remain incompletely characterized.

Notably, the elucidation of IGF2BP3’s regulatory role in ferroptosis provides new insights into potential therapeutic strategies for colon cancer. Recent studies have identified several regulators and inhibitors that can modulate IGF2BP3 expression. For instance, Xu et al. demonstrated that IGF2BP3 attenuates ferroptosis by maintaining m6A-methylated mRNAs encoding anti-ferroptotic factors (15). After screening a compound library of approximately 1800 FDA-approved small molecules, they found that Rigosertib could significantly reduce IGF2BP3 levels (15). Moreover, a recent study showed that the diazepine derivative JQ1 decreases IGF2BP3 expression, which in turn increases the survival rate of Ewing’s sarcoma patients (44). In the future, combination therapies involving traditional chemotherapy drugs and small molecule compounds could be used for drug-resistant colon cancer patients or to enhance therapeutic efficacy, especially in those with high IGF2BP3 expression. However, despite the established knowledge that IGF2BP3 is an RNA-binding protein that interacts with RNA through its KH domain to affect RNA metabolism, there is still a lack of research on the specific mechanisms of action. Consequently, the development of small molecules that selectively disrupt IGF2BP3-RNA interactions remains a challenge.

MicroRNAs (miRNAs) are potent genetic regulators, capable of influencing comprehensive cellular pathways through engagement with numerous target gene transcripts. Our study demonstrates that miR-98-5p directly targets IGF2BP3, thereby enhancing the sensitivity of cells to ferroptosis. Previous research had shown that miR-98-5p inhibits the proliferation of hepatocellular carcinoma (HCC) cells and induces apoptosis, at least partially, through the inhibition of its target gene IGF2BP1 (23). In head and neck squamous cell carcinoma, miR-98-5p suppresses tumor growth by downregulating IGF2BP2, which inhibits cell cycle progression while promoting apoptosis (32). Beyond the aforementioned mechanisms, which reduce the oncogenic effects of IGF2BP3 by influencing its interaction with RNA, the reduction of IGF2BP3 levels through miRNA can also inhibit tumor progression. Therefore, in the future, miRNA-based therapy could provide a potential therapeutic target for patients with high IGF2BP3 expression. However, the primary drawback of miRNA-based therapy remains the challenges of delivery efficiency and accurate targeting of the therapeutic miRNAs to the desired cells *in vivo* (45, 46).

In conclusion, our study reveals that IGF2BP3 regulates ferroptosis in colon cancer cells through SLC7A11, and miR-98-5p modulates ferroptosis in colon cancer cells by interacting with IGF2BP3. These findings provide new insights into potential combined treatment strategies for treating drug-resistant colon cancer patients or improving therapeutic outcomes. Overall, our findings hold substantial clinical applications and suggest that IGF2BP3 could be a promising candidate for therapeutic intervention to improve treatment efficacy in colon cancer.

## Data Availability

The datasets presented in this study can be found in online repositories. The names of the repository/repositories and accession number(s) can be found in the article/Supplementary Material. The high-throughput RNA-seq data is available in GEO database (GSE289758).
