# Supplementary material for: IGF2BP3 enhances ferroptosis resistance in colon cancer by stabilizing SLC7A11 and is regulated by miR-98-5p
Source: Front Oncol. 2025 Jun 3;15:1576895. doi: 10.3389/fonc.2025.1576895 (PMC12170320; doi:10.3389/fonc.2025.1576895)
Supplement: Supplementary file 1 [file Table1.docx]

| **Table S1 General characteristics of colon cancer patients for IHC analysis** | | | | |
| --- | --- | --- | --- | --- |
|  | **Low expression**  **(n=77)** | **High expression**  **(n=76)** | **X^2^/t value** | **P value** |
| Age(y)  Median(SD) | 65.44(12.03) | 66.13(11.13) | -0.368 | 0.713 |
| Sex  Male  Female | 43(55.8%)  34(44.2%) | 49(64.5%)  27(35.5%) | 1.188 | 0.276 |
| Tumor location  Right  Left | 40(51.9%)  37(48.1%) | 33(43.4%)  43(56.6%) | 1.115 | 0.291 |
| Alcohol consumption  No  Yes | 71(92.2%)  6(7.8%) | 66(86.8%)  10(13.2%) | 1.176 | 0.278 |
| Smoking  No  Yes | 60(77.9%)  17(22.1%) | 58(76.3%)  18(23.7%) | 0.056 | 0.813 |
| Hypertension  No  Yes | 44(57.1%)  33(42.9%) | 43(56.6%)  33(43.4%) | 0.005 | 0.944 |
| Diabetes  No  Yes | 60(77.9%)  17(22.1%) | 66(86.8%)  10(13.2%) | 2.094 | 0.148 |
| BMI(kg/m^2^)  BMI＜18.5  18.5≤BMI＜24  24≤BMI＜28  BMI≥28 | 8(10.4%)  36(46.8%)  24(31.2%)  9(11.7%) | 2(2.6%)  45(59.2%)  25(32.9%)  4(5.3%) | 6.844 | 0.077 |
| Abbreviations: IHC: Immunohistochemistry; SD: Standard deviation; BMI: Body mass index. | | | | |
