# Supplementary material for: IGF2BP3 enhances ferroptosis resistance in colon cancer by stabilizing SLC7A11 and is regulated by miR-98-5p
Source: Front Oncol. 2025 Jun 3;15:1576895. doi: 10.3389/fonc.2025.1576895 (PMC12170320; doi:10.3389/fonc.2025.1576895)
Supplement: Supplementary file 2 [file Table2.docx]

**Table S2 Oligonucleotides used in the experiments.**

| **Gene name** | **Sequence** | | **Application** |
| --- | --- | --- | --- |
| IGF2BP3 | F | 5′-ACTCCTCCCTACCCGCAGTTTG-3′ | primer |
|  | R | 5′-GATGATGGCACCGACTGATAGAGC-3′ |  |
| GAPDH | F  R | 5’-GTGGACCTGACCTGCCGTCTAG-3′  5’-GAGTGGGTGTCGCTGTTGAAGTC-3′ | primer |
| SLC7A11 | F  R | 5’-ACGGTGGTGTGTTTGCTGTCTC-3′  5’-GCTGGTAGAGGAGTGTGCTTGC-3′ | primer |
| sh-NC |  | 5′-TTCTCCGAACGTGTCACGT-3' | sh-RNA |
| sh-IGF2BP3 |  | 5'-GGCTCAGGGAAGAATTTAT-3’ | sh-RNA |
| Mimics NC | S  AS | 5’-UUCUCCGAACGUGUCACGUTT-3′  5’-ACGUGACACGUUCGGAGAATT-3′ | mimics |
| miR-98-5p | S  AS | 5'-UGAGGUAGUAAGUUGUAUUGUU-3'  5'-CAAUACAACUUACUACCUCAUU-3' | mimics |

Abbreviations: F: Forward; R: Reverse; S: Sense; AS: Antisense; NC: negative control; sh-RNA: Short hairpin-RNA
